# Initial red cell distribution width as a predictor of poor neurological outcomes in out-of-hospital cardiac arrest survivors in a prospective, multicenter observational study (the KoCARC study)

**DOI:** 10.1038/s41598-020-74842-y

**Published:** 2020-10-16

**Authors:** Seon Hee Woo, Woon Jeong Lee, Dae Hee Kim, Youngsuk Cho, Gyu Chong Cho

**Affiliations:** 1 Department of Emergency Medicine, Incheon St. Mary’s Hospital, College of Medicine, The Catholic University of Korea, #56 Dongsu-ro, Bupyeong-gu, Incheon, 21431 Republic of Korea; 2 Department of Emergency Medicine, Kang Dong Sacred Heart Hospital, Hallym University College of Medicine, Seoul, Republic of Korea

**Keywords:** Biomarkers, Medical research

## Abstract

This study aimed to investigate whether the initial red cell distribution width (RDW) at the emergency department (ED) is associated with poor neurological outcomes in out-of-hospital cardiac arrest (OHCA) survivors. We performed a prospective observational analysis of patients admitted to the ED between October 2015 and June 2018 from the Korean Cardiac Arrest Research Consortium registry. We included OHCA patients who visited the ED and achieved return of spontaneous circulation. Initial RDW values were measured at the time of the ED visit. The primary outcome was a poor neurological (Cerebral Performance Category, or CPC) score of 3–5. A total of 1008 patients were ultimately included in this study, of whom 712 (70.6%) had poor CPC scores with unfavorable outcomes. Higher RDW quartiles (RDW 13.6–14.9%, RDW ≥ 15.0%), older age, female sex, nonshockable initial rhythm at the scene, unwitnessed cardiac arrest, bystander cardiopulmonary resuscitation (CPR), medical history, low white blood cell counts and high glucose levels were associated with poor neurological outcomes in univariate analysis. In multivariate analysis, the highest RDW quartile was independently associated with poor neurological outcomes (odds ratio 2.04; 95% confidence interval 1.12–3.69; *p* = 0.019) at hospital discharge after adjusting for other confounding factors. Other independent factors including age, initial rhythm, bystander CPR and high glucose were also associated with poor neurological outcomes. These results show that an initial RDW in the highest quartile as of the ED visit is associated with poor neurological outcomes at hospital discharge among OHCA survivors.

## Introduction

Out-of-hospital cardiac arrest (OHCA) is becoming an issue worldwide, and a considerable amount of research is being conducted on factors related to survival^1–3^. Among patients with OHCA, return of spontaneous circulation (ROSC) occurs in a maximum of approximately 10%; furthermore, even OHCA survivors with ROSC often die in the emergency department (ED) within 24 h, and the survival-to-discharge rate at the hospital is known to be only approximately 10%. Additionally, many OHCA survivors show poor neurological outcomes at discharge^1,2^.

High red cell distribution width (RDW) values are used as a predictor of poor prognosis in many diseases, including cardiovascular diseases, perioperative stroke, malignancies and respiratory diseases^4–8^. Elevated RDW values are associated with adequate collateral development in patients with stable coronary artery disease and perioperative stroke in patients undergoing cardiac valve surgery^9,10^. Additionally, high RDW is associated with inflammatory conditions in various diseases, such as Hashimoto’s thyroiditis, rheumatoid arthritis, inflammatory bowel disease and gastrointestinal disorders^11–14^. In addition, a previous study found that RDW is strongly associated with prognostic factors reflecting severe inflammation, and changes in RDW within 72 h of ED admission are associated with 90-day mortality in critical patients with septic shock^15,16^. Moreover, Kim et al. reported that changes in the RDW value are an independent risk factor for all-cause mortality in OHCA patients^17^.

For the critical care management of OHCA survivors in EDs, the early prediction of neurological outcomes and mortality is also important for decision making by emergency medicine (EM) physicians in active and intensive care. Poor neurological outcomes in OHCA survivors are associated with ischemic reperfusion injury of the brain and can cause an increase in various inflammatory markers associated with sepsis-like physiological mechanisms of OHCA^18^. Studies indicate that C-reactive protein, procalcitonin, and the neutrophil-to-lymphocyte ratio can be used as inflammatory markers of cardiac arrest to predict neurologic outcomes^19–21^. However, few studies have used multicenter research data to investigate whether RDW is a prognostic factor predicting poor neurological outcomes^21^.

RDW can be easily checked from an initial blood sample in the ED, and the results can be obtained rapidly. Therefore, the aim of this study was to investigate whether initial RDW in the ED is associated with not only mortality but also poor neurological outcomes in OHCA survivors who visited the ED.

## Materials and methods

### Study design and setting

We conducted a multicenter prospective observational study based on the Korean Cardiac Arrest Research Consortium (KoCARC) registry between October 2015 and June 2018. The KoCARC is a multicenter collaborative research network of hospitals in the Republic of Korea^3^. The present study involved nontraumatic OHCA patients who were resuscitated. This study also included patients who had ROSC after cardiopulmonary resuscitation (CPR) by emergency medical services (EMS) on the scene. The exclusion criteria of this registry were patients with a history of terminal illness in their medical charts, patients who were pregnant, and patients with a previously documented “Do Not Resuscitate” order. The participating hospitals shared a standard registry form, and the investigators at each participating hospital collected EMS records and reviewed hospital medical charts for the clinical characteristics of the patients. The KoCARC registry was registered at clinicaltrials.gov as protocol NCT03222999.

### Study population and data collection

The study population included OHCA patients in the KoCARC registry who visited the ED and had ROSC at the scene or at the ED. We excluded OHCA patients under 15 years old and patients with incomplete or missing RDW data. We analyzed several variables from the KoCARC registry: patient demographic data (age and sex); initial prehospital rhythm; presence of witnesses to cardiac arrest; bystander CPR; prehospital ROSC; comorbidities (hypertension, diabetes mellitus, dyslipidemia); initial hospital rhythm; and white blood cells (WBCs), total bilirubin, glucose and RDW evaluated from the initial blood sample in the ED.

Neurological outcomes were measured with the Cerebral Performance Category (CPC) scale at hospital discharge. A CPC score of 1 (good cerebral performance) or 2 (moderate cerebral disability) was considered a favorable neurological outcome, and a score of 3 (severe cerebral disability), 4 (coma or vegetative state), or 5 (death) was considered a poor neurological outcome. Additionally, 24-h mortality and 30-day mortality in OHCA survivors were analyzed.

### Statistical analysis

We explored demographic characteristics, comorbidities and laboratory findings to analyze the predictors of poor outcomes. The Kolmogorov–Smirnov test was applied to continuous variables to test whether they followed the normal distribution; for those that were not normally distributed, the results are presented as the medians and interquartile ranges. RDW was divided into 4 quartiles, defined by ranges of ≤ 12.7%, 12.8–13.5%, 13.6–14.9% and ≥ 15.0%. Demographic and laboratory data of OHCA survivors were analyzed according to RDW quartiles, and the continuous variables were compared using the Kruskal–Wallis test. The categorical variables were compared and analyzed using the chi-square test or Fisher's exact test.

Univariate and multivariate Cox regression analyses were used for the prediction of 30-day mortality; they are presented as hazard ratios (HRs) and 95% confidence intervals (CIs). Multivariate logistic analysis was used to identify independent predictors of poor neurological outcome at hospital discharge. The predictors are presented with their odds ratios (ORs) and 95% CIs, and statistical significance is defined at a *p *value < 0.05. Receiver operating characteristic (ROC) curves were drawn to estimate the sensitivity and specificity of the RDW level in terms of predicting poor CPC; we also calculated the areas under the ROC curves (AUCs). All analyses were performed using SPSS software version 24.0 (SPSS, Inc., Chicago, IL, USA).

### Human ethical approval and informed consent

The KoCARC data collection protocol of this study was approved by the Institutional Review Boards (IRBs) of 34 participating hospitals. In a recent study, KoCARC investigators described the conceptualization, development, and implementation processes of the KoCARC registry to improve OHCA outcomes in the Republic of Korea^3^. The IRBs of most of the participating institutions (Seoul National University Hospital, Konkuk University Medical Center, Kyung Hee University Hospital, Korea University Guro Hospital, Korea University Anam Hospital, SMG-SNU Boramae Medical Center, Yonsei University Severance Hospital, Yonsei University Gangnam Severance Hospital, Hallym University Kangdong Sacred Heart Hospital, Hallym University Kangnam Sacred Heart Hospital, Hanyang University Seoul Hospital, Kyungpook National University Hospital, Chosun University Hospital, Seoul National University Bundang Hospital, Myongji Hospital, Korea University Ansan Hospital, Dongguk University Ilsan Hospital, Bundang Jesaeng Hospital, Wonkwang University Sanbon Hospital, Hallym University Dongtan Sacred Heart Hospital, Chungbuk National University Hospital, Soonchunhyang University Cheonan Hospital, Jeju National University Hospital, Hanyang University Guri Hospital, Gyeongsang National University Hospital, Ajou University Hospital, Pusan National University Yangsan Hospital, Ewha Womans University Mokdong Medical Center, Inha University Hospital, and Hallym University Sacred Heart Hospital) waived the requirement for informed consent^3^. However, Samsung Medical Center, Asan Medical Center, Wonju Severance Christian Hospital and Incheon St. Mary’s Hospital received informed consent from individual participants for the follow-up survey of neurological outcomes and death. (The requirement for informed consent was waived for participants who did not need a telephone interview for the follow-up survey.) This study was approved by the Ethics Committee of Incheon St. Mary’s Hospital (OC15OIMI0134). All methods were performed in accordance with the relevant guidelines and regulations.

## Results

### Characteristics of the study population

**Figure 1:**
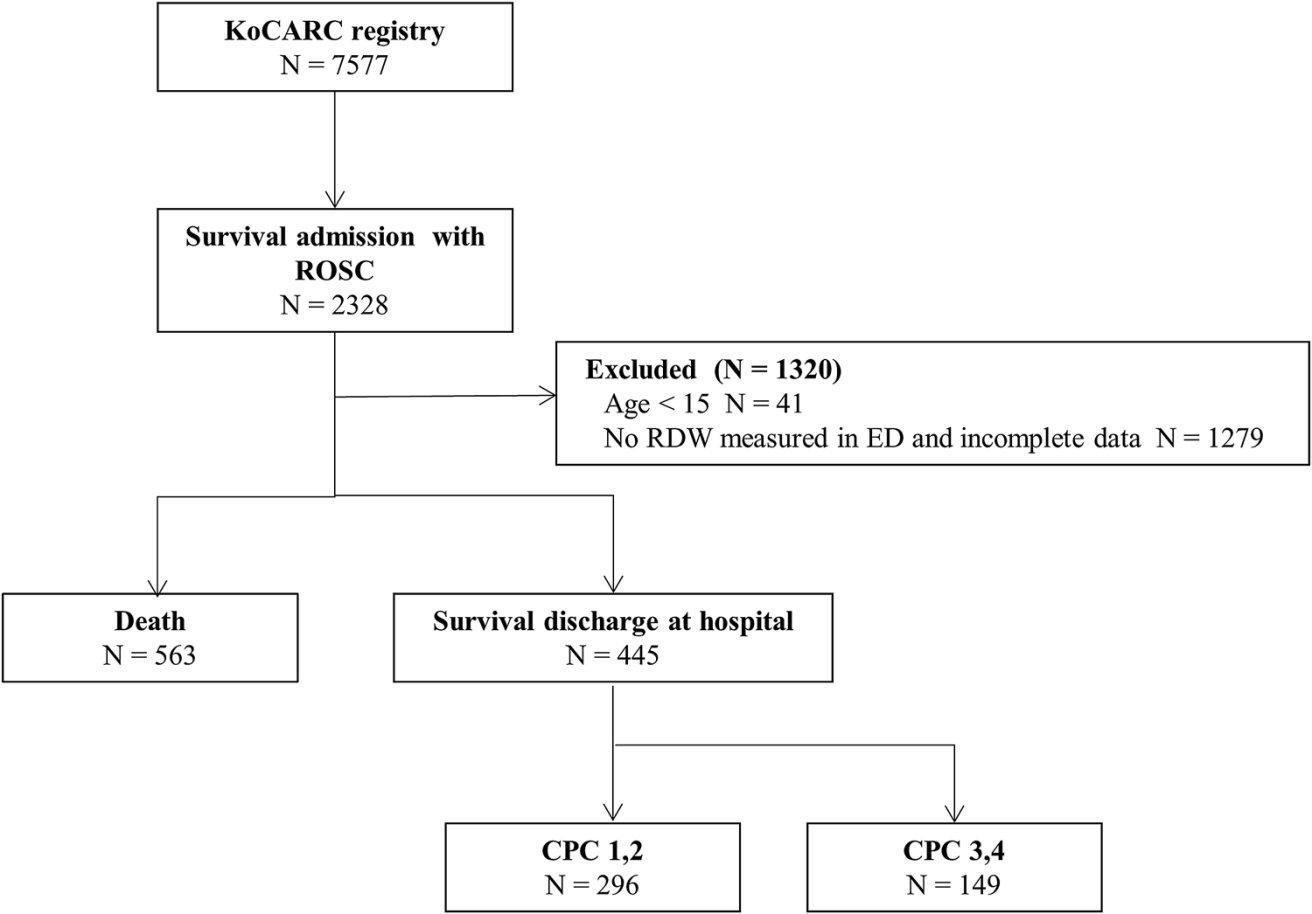
Flow diagram of out-of-hospital cardiac arrest survivors in this study.

Of the 7577 OHCA patients examined during this research period, 5249 were excluded because they did not survive to hospital admission. In addition, of the 2328 patients who survived admission, 41 patients were excluded because they were under 15 years old. Moreover, 1279 patients were excluded due to incomplete data or missing initial RDW at the ED. We ultimately included a total of 1008 nontraumatic OHCA survivors (Fig. 1).

The mean age of the patients was 61.9 (SD 15.3) years, the median (IQR) age was 62 (52, 74) years, and 717 (71.1%) patients were male. Two hundred and fifty-three patients (25.1%) had high RDW values (≥ 15%) on arrival in the ED. After ROSC, a total of 285 (28.3%) patients died within 24 h, and 550 (54.6%) patients died within 30 days. A total of 712 (70.6%) patients had poor CPC scores (indicating unfavorable outcomes) at hospital discharge (Table 1). The clinical characteristics of the patients according to their initial RDW quartiles after ROSC were analyzed and are shown in Table 2. The factors that significantly differed among the four RDW quartile groups included age, initial shockable rhythm at the scene, bystander CPR, prehospital ROSC, history of diabetes, total bilirubin and glucose. Additionally, 24-h mortality, 30-day mortality and poor CPC outcomes at hospital discharge were significantly different across the four RDW quartiles (*p* < 0.001, *p* < 0.001, *p* < 0.001) (Table 2).

**Table 1:** Baseline characteristics of the study population.

	Total
	N = 1008
**Age (years)**	
Mean ± SD	61.9 ± 15.3
Median (IQR)	62 (52, 74)
**Sex**	
Male	717 (71.1)
Female	291 (28.9)
**Initial shockable rhythm at the scene**	
Nonshockable	552 (59.9)
Shockable	369 (40.1)
**Initial shockable rhythm in the hospital**	
Nonshockable	927 (92)
Shockable	81 (8)
**Bystander CPR**	
No	423 (45.9)
Yes	499 (54.1)
**Prehospital ROSC**	
No	616 (61.1)
Yes	392 (38.9)
**Hypertension**	
No	490 (53.5)
Yes	426 (46.5)
**Diabetes mellitus**	
No	636 (70.6)
Yes	265 (29.4)
**Dyslipidemia**	
No	814 (93.2)
Yes	59 (6.8)
**Witnessed arrest**	
No	241 (24.5)
Yes	742 (75.5)
**RDW (quartile)**	
< 25% (≤ 12.7%)	238 (23.6)
25–50% (12.8–13.5%)	243 (24.1)
50–75% (13.6–14.9%)	274 (27.2)
> 75% (≥ 15%)	253 (25.1)
**White blood cell count (10**^**9**^**/L)**	
Mean ± SD	13 ± 5.6
Median (IQR)	12 (9.3, 15.5)
**Total bilirubin (mg/dL)**	
Mean ± SD	0.7 ± 1.2
Median (IQR)	0.5 (0.3, 0.7)
**Glucose (mg/dL)**	
Mean ± SD	279.4 ± 143.9
Median (IQR)	264 (190, 339)
**Survival discharge**	
No	563 (55.9)
Yes	445 (44.2)
**24-h mortality**	
No	723 (71.7)
Yes	285 (28.3)
**30-day mortality**	
No	458 (45.4)
Yes	550 (54.6)
**Poor CPC**	
No	296 (29.4)
Yes	712 (70.6)

*CPR* cardiopulmonary resuscitation, *RDW* red cell distribution width, *ROSC* return of spontaneous circulation, *CPC* Cerebral Performance Category.

**Table 2:** Patient characteristics stratified according to red cell distribution width quartiles.

	≤ 12.7%	12.8–13.5%	13.6–14.9%	≥ 15%	*p *value
	n = 238	n = 243	n = 274	n = 253	
**Age**					< 0.001
Median (IQR)	57.5 (49, 69)	61 (51, 73)	65 (55, 76)	66 (55, 76)	
**Sex**					0.075
Male	178 (74.8)	180 (74.1)	194 (70.8)	165 (65.2)	
Female	60 (25.2)	63 (25.9)	80 (29.2)	88 (34.8)	
**Initial shockable rhythm at the scene**					< 0.001
Nonshockable	111 (51.4)	115 (52.5)	150 (59.8)	176 (74.9)	
Shockable	105 (48.6)	104 (47.5)	101 (40.2)	59 (25.1)	
**Initial shockable rhythm in the hospital**					0.076
Nonshockable	219 (92)	223 (91.8)	244 (89.1)	241 (95.3)	
Shockable	19 (8)	20 (8.2)	30 (11)	12 (4.7)	
**Witnessed arrest**					0.306
No	68 (29.1)	55 (23.1)	64 (23.9)	54 (22.2)	
Yes	166 (70.9)	183 (76.9)	204 (76.1)	189 (77.8)	
**Bystander CPR**					< 0.001
No	84 (37.7)	86 (39.5)	126 (50.2)	127 (55.2)	
Yes	139 (62.3)	132 (60.6)	125 (49.8)	103 (44.8)	
**Prehospital ROSC**					< 0.001
No	136 (57.1)	118 (48.6)	172 (62.8)	190 (75.1)	
Yes	102 (42.9)	125 (51.4)	102 (37.2)	63 (24.9)	
**Hypertension**					0.078
No	128 (59.3)	125 (56.6)	124 (49.4)	113 (49.6)	
Yes	88 (40.7)	96 (43.4)	127 (50.6)	115 (50.4)	
**Diabetes mellitus**					< 0.001
No	170 (79.1)	167 (77.3)	168 (68.6)	131 (58.2)	
Yes	45 (20.9)	49 (22.7)	77 (31.4)	94 (41.8)	
**Dyslipidemia**					0.390
No	199 (93.9)	192 (91)	227 (95)	196 (92.9)	
Yes	13 (6.1)	19 (9)	12 (5)	15 (7.1)	
**Witnessed arrest**					0.306
No	68 (29.1)	55 (23.1)	64 (23.9)	54 (22.2)	
Yes	166 (70.9)	183 (76.9)	204 (76.1)	189 (77.8)	
**WBC count (10**^**9**^**/L)**					0.053
Median (IQR)	12.3 (9.8, 15.5)	12.4 (9.5, 15.5)	12.2 (9.6, 16.1)	11.3 (8.6, 15.3)	
**Total bilirubin (mg/dL)**					< 0.001
Median (IQR)	0.5 (0.3, 0.6)	0.5 (0.3, 0.7)	0.4 (0.3, 0.7)	0.6 (0.3, 1)	
**Glucose (mg/dL)**					< 0.001
Median (IQR)	285 (214, 346)	267 (209, 342)	265.5 (193, 350)	234 (150, 321)	
**Survival discharge**					< 0.001
No	117 (49.2)	108 (44.4)	158 (57.7)	180 (71.2)	
Yes	121 (50.8)	135 (55.6)	116 (42.3)	73 (28.9)	
**24-h mortality**					< 0.001
No	190 (79.8)	178 (73.3)	198 (72.3)	157 (62.1)	
Yes	48 (20.2)	65 (26.8)	76 (27.7)	96 (37.9)	
**30-day mortality**					< 0.001
No	124 (52.1)	139 (57.2)	120 (43.8)	75 (29.6)	
Yes	114 (47.9)	104 (42.8)	154 (56.2)	178 (70.4)	
**Poor CPC**					< 0.001
No	85 (35.7)	103 (42.4)	72 (26.3)	36 (14.2)	
Yes	153 (64.3)	140 (57.6)	202 (73.7)	217 (85.8)	

Values are shown as the number(percentage) for categorical variables and the mean (SD) or median (IQR) for other variables.

*p* values are calculated using the chi-square test for categorical variables and the Kruskal–Wallis test for continuous variables.

*CPR* cardiopulmonary resuscitation, *RDW* red cell distribution width, *ROSC* return of spontaneous circulation, *WBC* white blood cell, *CPC* Cerebral Performance Category.

### Prediction of 30-day mortality in OHCA survivors

Regarding 30-day mortality, univariate analysis showed that the predictive factors included RDW in the highest quartile group, older age, female sex, initial rhythm at the scene, witnesses to cardiac arrest and a history of diabetes; moreover, WBC count and glucose level were also found to be predictive factors. In the multivariate analysis, RDW in the highest quartile (≥ 15.0%) was shown to be a predictive factor of 30-day mortality (HR 1.39; 95% CI 1.05–1.82; *p* = 0.020) (Table 3). Other significant factors included age, initial rhythm at the scene, WBC count, and glucose level (*p* < 0.001, *p* < 0.001, *p* = 0.039 and *p* = 0.016).

**Table 3:** Hazard ratios of risk factors for mortality within 30 days.

	Univariate hazard ratio (95% CI)	*p *value	Multivariate hazard ratio (95% CI)	*p *value
**RDW (quartile)**				
< 25% (≤ 12.7%)	(reference)		(reference)	
25–50% (12.8–13.5%)	0.91 (0.70–1.19)	0.483	0.92 (0.68–1.24)	0.564
50–75% (13.6–14.9%)	1.26 (0.99–1.60)	0.063	1.13 (0.85–1.48)	0.402
> 75% (≥ 15%)	1.73 (1.36–2.18)	< 0.001	1.39 (1.05–1.82)	0.020
Age	1.02 (1.02–1.03)	< 0.001	1.02 (1.01–1.02)	< 0.001
Female	1.47 (1.24–1.76)	< 0.001	1.13 (0.93–1.39)	0.225
Initial shockable rhythm at the scene	0.36 (0.29–0.44)	< 0.001	0.46 (0.36–0.58)	< 0.001
Hypertension	1.18 (0.98–1.40)	0.074		
Diabetes mellitus	1.43 (1.19–1.73)	< 0.001	1.14 (0.93–1.39)	0.225
Dyslipidemia	0.74 (0.50–1.10)	0.131		
Witness arrest	0.74 (0.61–0.89)	0.002	0.84 (0.68–1.04)	0.106
Bystander CPR	0.85 (0.72–1.02)	0.078		
WBC count (10^9^/L)	0.98 (0.96–0.99)	0.004	0.98 (0.96–1.00)	0.039
Total bilirubin (mg/dL)	1.02 (0.95–1.09)	0.597		
Glucose (mg/dL)	1.00 (1.00–1.00)	0.009	1.00 (1.00–1.00)	0.016

Hazard ratios were calculated by using univariate and multivariate Cox regression analysis.

*RDW* red cell distribution width, *CPR* cardiopulmonary resuscitation, *WBC* white blood cell.

### Prediction of poor neurological outcomes in OHCA survivors

In the univariate analysis, RDW values in the ranges of 13.6–14.9% and ≥ 15.0% were associated with CPC scores indicating poor neurological outcomes at hospital discharge (*p* = 0.021, *p* < 0.001). After adjustment for demographic, prehospital and laboratory findings with *p* values < 0.05 in univariate analysis, multivariate logistic analysis was performed. Of all the independent prognostic factors, RDW ≥ 15% at ED admission had the highest OR for poor neurological outcomes at ED admission (OR 2.04, 95% CI 1.12–3.69, *p* = 0.019) (Table 4).

**Table 4:** Odds ratios for poor Cerebral Performance Category at hospital discharge.

	Univariate analysis (95% CI)	*p *value	Multivariate analysis (95% CI)	*p *value
**RDW (quartile)**				
< 25% (≤ 12.7%)	(reference)		(reference)	
25–50% (12.8–13.5%)	0.76 (0.52–1.09)	0.134	0.60 (0.35–1.01)	0.053
50–75% (13.6–14.9%)	1.56 (1.07–2.27)	0.021	1.32 (0.78–2.24)	0.308
> 75% (≥ 15%)	3.35 (2.15–5.21)	< 0.001	2.04 (1.12–3.69)	0.019
RDW (value)	1.03 (1.00–1.06)	0.082		
Age	1.05 (1.04–1.06)	< 0.001	1.04 (1.02–1.05)	< 0.001
Female	2.30 (1.65–3.22)	< 0.001	1.42 (0.89–2.25)	0.138
Initial shockable rhythm at the scene	0.09 (0.06–0.12)	< 0.001	0.12 (0.08–0.18)	< 0.001
Hypertension	1.45 (1.09–1.92)	0.011	0.82 (0.53–1.27)	0.383
Diabetes mellitus	2.46 (1.74–3.50)	< 0.001	1.45 (0.89–2.37)	0.135
Dyslipidemia	0.48 (0.28–0.81)	0.006	0.50 (0.24–1.06)	0.071
Witness arrest	0.53 (0.37–0.75)	< 0.001	0.70 (0.44–1.13)	0.149
Bystander CPR	0.57 (0.43–0.76)	< 0.001	1.55 (1.03–2.34)	0.037
WBC count (10^9^/L)	0.98 (0.95–1.00)	0.044	0.97 (0.94–1.01)	0.123
Total bilirubin (mg/dL)	1.00 (0.89–1.12)	0.997		
Glucose (mg/dL)	1.00 (1.00–1.00)	< 0.001	1.00 (1.00–1.01)	< 0.001

*RDW* red cell distribution width, *CPR* cardiopulmonary resuscitation, *WBC* white blood cell.

Odds ratios were calculated by logistic regression analysis.

**Figure 2:**
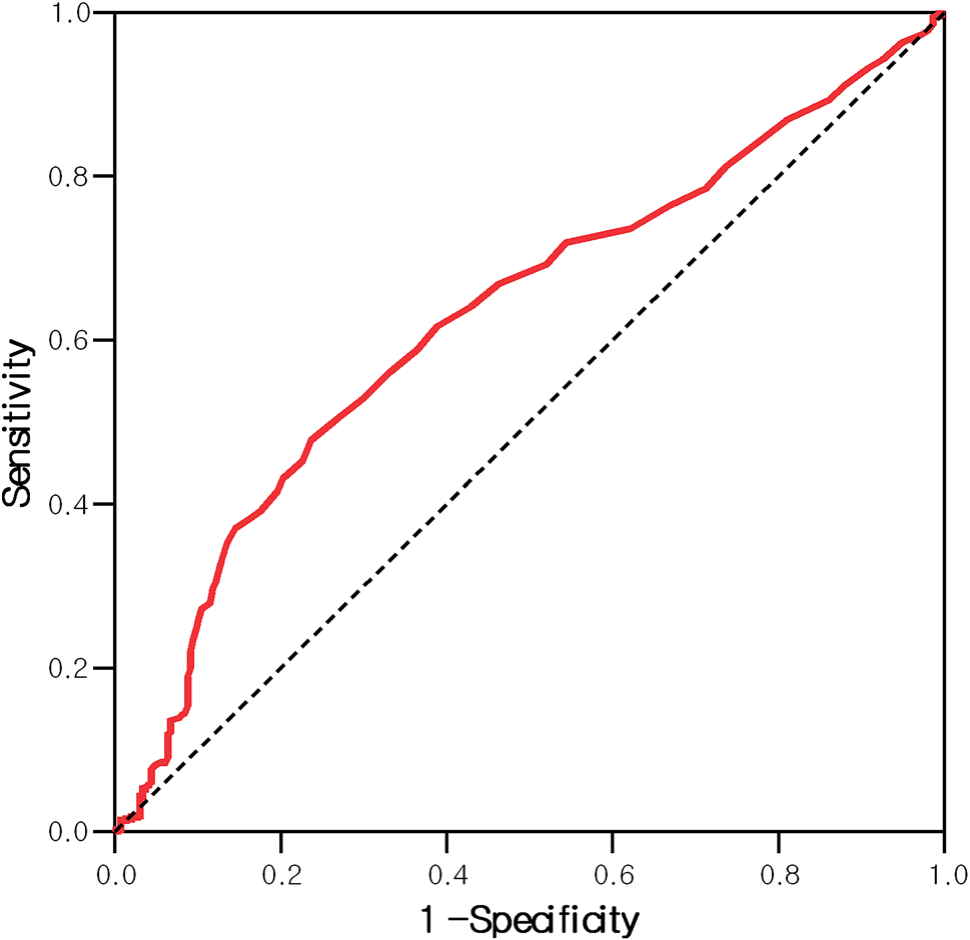
Receiver operating characteristic curve of the initial red cell distribution width for the prediction of poor neurological outcomes.

RDW at the ED after ROSC had an AUC of 0.630 (95% CI 0.593–0.666) for predicting poor CPC at hospital discharge (*p* < 0.001). The group with the highest RDW (≥ 15.0%) showed a sensitivity of 29.8%, a specificity of 88.2%, a positive predictive value (PPV) of 85.8% and a negative predictive value (NPV) of 34.3% for poor CPC outcomes (Fig. 2).

## Discussion

The present study showed that RDW ≥ 15% was an independent predictor of poor CPC scores at hospital discharge in OHCA survivors. Significant predictive factors other than RDW included age and initial rhythm at the scene. RDW was an independent marker predicting 30-day mortality and poor neurological outcomes.

Ideal prognostic markers for use in the ED should be inexpensive and rapidly available, with rapidly confirmable results, to facilitate the decision making of ED physicians about the critical care of OHCA patients. RDW, a measurement of the range of variation in erythrocyte size and volume, can be obtained from a complete blood count from routine laboratory testing at the ED^22^. RDW levels may be increased or decreased according to OHCA patients’ initial clinical conditions at the ED^17,21^. In a previous study, RDW was an independent risk factor predicting the severity and progression of cardiovascular disease, inflammation and oxidative stress^23–29^. The pathophysiology of the association between the outcome of cardiovascular disease and RDW is unclear. However, elevated RDW may be related myocardial ischemia, as heart failure caused by atherosclerosis of the coronary vessel walls is stimulated by inflammation^9,23,24^. Some studies have shown that RDW is an effective prognostic factor for outcomes in critically ill patients and septic patients^28–32^. In Tonelli’s study, RDW levels were associated with the risk of adverse outcomes and mortality in patients with coronary disease^33^. Felker’s study showed an association between increased RDW and mortality in congestive heart failure (CHF) patients (HR 1.17)^24^. Additionally, among 219 OHCA patients who had ROSC, the highest RDW quartile (> 15.4%) in OHCA patients admitted to the ED was independently associated with 30-day all-cause mortality (HR 1.95)^17^. Our study yielded similar results. Among the 1008 OHCA patients who had ROSC, the highest RDW (≥ 15.0%) quartile in admitted ED patients was independently associated with an increased risk of mortality during the 30-day period (HR 1.39). Additionally, RDW showed significantly elevated values in subarachnoid hemorrhage (SAH) patients and cerebral infarction patients with poor outcomes^34,35^. Although cardiovascular disease is known to be the main cause of OHCA, stroke may also be a cause. Studies on the association between increased RDW and poor outcomes in cardiovascular disease and stroke have already been proposed^23–26,34,35^. Therefore, the poor outcome of OHCA patients may be related to RDW.

Few studies have focused on the association between RDW and neurological outcomes in patients with ROSC following OHCA^21^. In a previous single-center study, high RDW levels on admission were associated with poor CPC outcomes (scores of 3–5) among cardiac arrest (CA) survivors admitted to the intensive care unit (390 patients)^21^. They showed that an RDW threshold of 13.4% on admission had a sensitivity of 64% and a specificity of 43% for predicting poor CPC (3–5) after 3 months of survival^21^. The present study showed that an initial RDW value of ≥ 15.0% at the time of the ED visit had a sensitivity of 29.8% and a specificity of 88.2% for predicting unfavorable outcomes at hospital discharge. Although the sensitivity of RDW in predicting neurological outcomes is not high, it showed relatively high specificity for predicting poor CPC scores in the ED. RDW combined with other prognostic factors may be useful in predicting the neurological prognosis of OHCA survivors in the ED. Our investigation is a multicenter study that is based on ED visits. The study included all patients who died within 24 h after ROSC, and we analyzed the prediction of poor CPC at hospital discharge. This may be why the present study has higher specificity than previous studies.

RDW is measured as part of a complete blood count, which is a standard routine laboratory test in the ED^22^; thus, RDW is easily obtainable and simple for EM physicians to use as a prognostic marker. However, the direct association between the prognostic variable of RDW and the outcome variable of CA is unclear. The presence of increased RDW in OHCA survivors with poor outcomes may be mediated by other factors rather than a single prognostic factor. A previous study reported the predictive value of RDW and N-terminal pro-brain natriuretic peptide (NT-proBNP) for the mortality of CHF patients. The authors indicated that the RDW value was a predictor of mortality but had lower predictive value than NT-proBNP. They suggested that RDW be used along with other biomarkers as a complementary prognostic factor to improve the accuracy of prognosis^25^. Therefore, further prospective studies on OHCA survivors are needed to validate the predictive association between RDW and poor outcomes.

This study has several limitations. First, our study has some selection bias. Although prospectively collected KoCARC registry data were analyzed, 1279 patients were excluded from the analysis because of incomplete or missing data, as their RDW values had not been measured in the ED. Second, we could not analyze the relationship between RDW and long-term neurological outcomes. Further studies should examine 6-month neurological outcomes, activities of daily living, and return to work as elements of health-related quality of life in OHCA survivors. Additionally, although this study included only OHCA survivors who met the inclusion criteria, there are various confounding factors because the baseline characteristics of each CA patient are different. However, this study is meaningful because it suggested that RDW could be used as an early prognostic marker for poor neurological outcomes in OHCA survivors.

In conclusion, high initial RDW values in the ED were associated with poor neurological outcomes at hospital discharge and with 30-day mortality in OHCA survivors. Thus, these results suggest that initial RDW can be a useful predictive marker of poor outcomes in OHCA survivors who visit the ED.

## Data Availability

All data used to support the findings of this study are available from the corresponding author upon request.
